# Characterization of Two Cryptic Plasmids Isolated in Haiti from Clinical *Vibrio cholerae* Non-O1/Non-O139

**DOI:** 10.3389/fmicb.2017.02283

**Published:** 2017-11-23

**Authors:** Daniela Ceccarelli, Geneviève Garriss, Seon Y. Choi, Nur A. Hasan, Ramunas Stepanauskas, Mihai Pop, Anwar Huq, Rita R. Colwell

**Affiliations:** ^1^Department of Cell Biology and Molecular Genetics, Maryland Pathogen Research Institute, University of Maryland, College Park, MD, United States; ^2^Department of Bacteriology and Epidemiology, Wageningen Bioveterinary Research, Lelystad, Netherlands; ^3^Department of Microbiology, Cell Biology and Tumor Biology, Karolinska Institutet, Stockholm, Sweden; ^4^CosmosID Inc., Rockville, MD, United States; ^5^University of Maryland Institute for Advanced Computer Studies, University of Maryland, College Park, MD, United States; ^6^Bigelow Laboratory for Ocean Sciences, East Boothbay, ME, United States; ^7^Maryland Institute of Applied Environmental Health, University of Maryland, College Park, MD, United States; ^8^Department of Environmental Health Sciences, Bloomberg School of Public Health, Johns Hopkins University, Baltimore, MD, United States

**Keywords:** *Vibrio cholerae*, non-O1/non-O139, Haiti, plasmid, cholera

## Abstract

We report the complete sequence of two novel plasmids, pSDH-1 and pSDH-2, isolated from clinical *Vibrio cholerae* non-O1/non-O139 during the early phase of the 2010 Haitian cholera epidemic. Plasmids were revealed by employing single-cell genomics and their genome content suggests self-mobilization and, for pSDH-2, a toxin-antitoxin (TA) system for plasmid stabilization was identified. The putative origin of replication of pSDH-2 was mapped suggesting it replicates following the ColE1 model of plasmid replication. pSDH-1 and pSDH-2 were widespread among environmental *V. cholerae* non-O1/non-O139 with variable prevalence in four Haitian Departments. pSDH-2 was the most common element, either alone or with pSDH-1. The two plasmids detection adds to the composite scenario of mobile genetic elements (MGEs) observed in *V. cholerae* in Haiti. The role these small cryptic plasmids circulating in *Vibrio* spp. play in bacterial fitness or pathogenicity merits further investigation.

## Introduction

In January 2010, Haiti was struck by a devastating earthquake responsible of a humanitarian crisis still ongoing. Since the first case was diagnosed in October 2010 (World Health Organization, 2010) cholera remains a serious health threat in Haiti. 41,421 new cases were reported by the WHO for Haiti alone in 2016 (World Health Organization, 2017), a reminder of how significant the epidemic still is. The agent of epidemic cholera was recognized as *Vibrio cholerae* toxigenic serogroup O1 (Ceccarelli et al., 2011; Chin et al., 2011) although a population of *V. cholerae* non-O1/non-O139 was shown to coexist with clinical *V. cholerae* O1 in Haiti early in the epidemic (Hasan et al., 2012). *V. cholerae* non-O1/non-O139 have been isolated in Haiti, mostly from the aquatic environment (Baron et al., 2016), but proof of their presence in stools collected from asymptomatic Haitian infants two years prior to the earthquake has been published (Liu et al., 2014). *V. cholerae* non-O1/non-O139 are natural inhabitants of estuarine and coastal waters and, as opportunistic pathogens, can be responsible for infections other than cholera, generally through the consumption of raw or undercooked seafood. Non-O1/non-O139 *V. cholerae* infections are continuously reported worldwide (Chomvarin et al., 2014; Crowe et al., 2016; Hirk et al., 2016), emphasizing their growing clinical significance.

Mobile genetic elements (MGEs) play an essential role in gene transfer. The high incidence of plasmids in marine Vibrios (Hazen et al., 2007; Zhang et al., 2012; Aedo et al., 2014; Wang et al., 2016) suggests that the marine environment is an important source of genome plasticity mediated by acquisition of MGEs and recombination. The Haitian environment is no exception and *V. cholerae* non-O1/non-O139 has shown a higher genomic variability compared to clinical *V. cholerae* O1. We previously reported the presence of the SXT/R391-related integrating conjugative element ICE*Vch*Hai2 circulating among closely related *V. cholerae* non-O1/non-O139 (Ceccarelli et al., 2013), carrying new genes involved in recombination. More recently, we identified MGI*Vch*Hai6, a novel mobilizable genomic island (MGI) containing a mercury resistance transposon and an integron conferring resistance to chloramphenicol, trimethoprim and streptomycin/spectinomycin (Carraro et al., 2016). MGI*Vch*Hai6, whose sibling MGIs have also been detected *in silico* in the Indian subcontinent, North and South America, can be mobilized by IncA/C plasmids, highly conjugative elements also known to circulate in Haitian *V. cholerae* (Folster et al., 2014).

While performing genomic heterogeneity tests on Haitian clinical strain *V. cholerae* HC-1A2, using single cell genomics techniques (Stepanauskas, 2012), we unexpectedly recovered two novel plasmids. Complete sequence and distribution of these plasmids in clinical and environmental *V. cholerae* non-O1/non-O139 and O1 isolated during the early days of the 2010 epidemic in Haiti are here reported, adding up to the composite scenario of MGEs circulating in Haitian *V. cholerae*.

## Materials and methods

### Plasmid sequencing and annotation

*V. cholerae* non-O1/non-O139 strain HC-1A2 was isolated from stool sample of a cholera patient in Saint-Marc (Artibonite) in 2010, with traditional methods of isolation and identification as previously described (Hasan et al., 2012). 317 single amplified genomes (SAGs) of *V. cholerae* HC-1A2 were generated at the Bigelow Laboratory Single Cell Genomics Center (scgc.bigelow.org), as previously described (Swan et al., 2013). Briefly, single cell DNA genome sequencing involves isolating a single cell and performing whole-genome-amplification; this step is then followed by construction of sequencing libraries and DNA sequencing by a next-generation sequencer. One of the SAGs of *V. cholerae* HC-1A2, AD-538-E13, was genomically sequenced as follows. Single cell multiple displacement amplification products were sheared with M220 Focused Ultrasonicator (Covaris, Woburn, MA, USA) and 450–550 bp fragments were size-selected with BluePippin (Sage Science, Beverly, MA, USA). Paired-end sequencing libraries were generated using NEBNext Ultra DNA Library Prep kit (New England Biolabs, Ipswich, MA, USA), and 14 million 2 × 250 bp reads were produced using MiSeq (Illumina, San Diego, CA). The obtained sequence reads were quality-trimmed with Trimmomatic v0.32 5 using the following settings: -phred33 LEADING:0 TRAILING:5 SLIDINGWINDOW:4:15 MINLEN:36. Human DNA (≥95% identity to *H. sapiens* reference assembly GRCh38) and low complexity reads (containing <5% of any nucleotide) were removed. The quality-filtered reads were digitally normalized with kmernorm 1.05 (http://sourceforge.net/projects/kmernorm) using settings -k 18 -t 80 -c 2 and de novo assembled with Geneious 7.1.2 (Biomatters, Auckland, New Zealand) using medium-low sensitivity and allowing for circularization. Circular contigs with no homology to the previously sequenced *V. cholerae* HC-1A2 genome (Hasan et al., 2012) were selected for further analysis. The annotation of these putative plasmids was performed using RAST and GLIMMER. RAST subsystem for functional annotation was used to determine position 1 of the assembled plasmids. Database searches were carried out through NCBI using ORF Finder, BLASTn, BLASTp, and SMART (Schultz et al., 1998; Letunic et al., 2015).

### Bacterial isolates

By the end of October 2010 cholera had been confirmed in four of Haiti's 10 departments (administrative regions): Artibonite, Centre, Nord and Ouest, including the capital Port-au-Prince, and the capital's Cité Soleil district. In November 2010 sampling in these four Haitian departments took place; sampling details and isolation procedures are described elsewhere (Hasan et al., 2012). 175 Haitian bacterial strains isolated during this sampling campaign were screened for presence of plasmids pSDH-1 and pSDH-2: 70 clinical *V. cholerae* O1, 50 clinical *V. cholerae* non-O1/non-O139, 22 environmental *V. cholerae* non-O1/non-O139 and 33 isolates of *Vibrio* spp. and *Aeromonas* spp. Antiserum kits for *V. cholerae* O1 (*V. cholerae* Antiserum Poly; Difco, USA) and *V. cholerae* O139 (O139 Bengal; Hardy Diagnostics, USA) were used to determine serotype by slide agglutination, according to manufacturers' instructions. Serotyping was confirmed by multiplex PCR (Hoshino et al., 1998). Bacterial isolates were stored at −80°C in LB broth containing 50% (vol/vol) glycerol.

### DNA extraction and PCR amplification

Genomic DNA was extracted by boiling method as previously described (Ausubel et al., 1990). PCR assays were performed employing two sets of primers per plasmid designed using Primer3 (http://www.simgene.com/Primer3): set 1 (p1_cds1-Fw: AACAGATGGCGCAATCATAA; p1_cds5- Rev: CGCTCGTCAATCGTCCTATG) and set2 (p1_inter-Fw: TGAATTAAGCCCGTTGGACT; p1_cds7-Rev: CAATTAAACATCCTGACTTTGAAAAA) for pSDH-1; set3 (p2_inter-Fw: ACAAGGCTAGCCCACCTGTA; p2_cds1-Rev: ATTAGCAGCTGCGCGATTAG) and set4 (p2_cds4-Fw: GGTTGGGTCATTTGTTCCAT; p2_cds6-Rev: AGTGTCCCTCATCGAAATGC) for pSDH-2. Amplicon sizes were 1,671, 1,112, 1,110, and 1,495 bp, respectively. Thermal cycling conditions were as follows: (i) 5 min at 95°C; (ii) 35 cycles of 30 s at 95°C, 30 s at 64°C, and 2 min at 72°C; and (iii) 5 min at 72°C. PCR was performed in a 25-μl reaction mix containing 12.5 μl of GoTaq master mix polymerase (Promega). PCR amplicons were purified using a QIAquick PCR purification kit (Qiagen) according to manufacturer's instructions, and DNA sequences were determined by Eurofins Genomics (Huntsville, AL, USA).

### Nucleotide sequence accession numbers

Plasmid sequences have been submitted to the GenBank database with accession numbers: KY486775 (pSDH-1) and KY486774 (pSDH-2).

## Results and discussion

**Figure 1:**
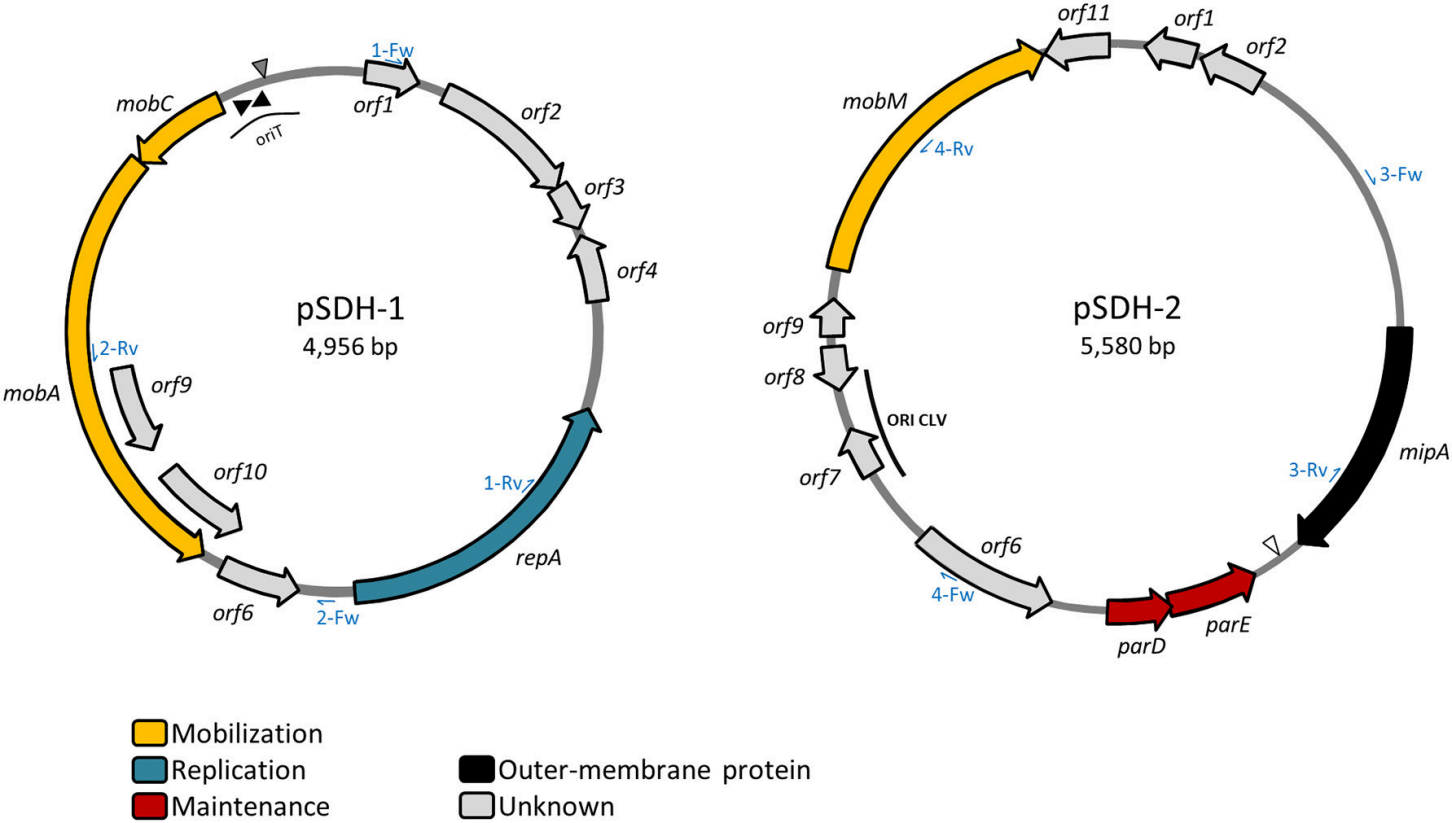
Schematic representation of plasmids pSDH-1 and pSDH-2. Predicted open reading frames (ORFs) are indicated by arrows pointing in the direction of transcription. ORFs to which a putative function could be assigned are color-coded. Size of the plasmids is indicated. Position of the inverted repeats of the putative oriT region of pSDH-1 are indicated by black triangles. Location of the putative *nic* site is indicated by a gray triangle. The open triangle indicates the position of the putative *xer* recombination site of pSDH-2. The position of the putative CLV-like origin of replication of pSDH-2 is indicated. Position of PCR primers used for screening for the presence of both plasmids are indicated by blue half-arrows and are labeled with the number of the set followed by Fw (forward) or Rv (reverse).

Single-cell genomics performed on the pure culture of strain *V. cholerae* HC-1A2 with the aim of performing genomic heterogeneity tests revealed the presence of two previously unknown, circular plasmids pSDH-1 (4,985 bp) and pSDH-2 (5,580 bp) (Figure 1). We hypothesize that these two plasmids were likely missed during the original sequencing of *V. cholerae* isolates (Hasan et al., 2012) due to the shotgun library construction step (with ~3 kb insert).

### Identification and genomic structure of plasmid pSDH-1

We found that plasmid pSDH-1 encodes 10 putative open reading frames (ORFs) (Figure 1 and Table 1). *orf1* to *orf4* and *orf6* encode putative proteins of unknown function for which no known homologs could be found. BlastP search revealed that the predicted protein encoded by *orf5* shows 45% identity to a replicase family protein from plasmid pRGRH0347, and contains replicase (RepA) and priCT_1 (primase C-terminal) domains. It is possible that this protein is involved in replication of pSDH-1. Accordingly, *orf5* was renamed *repA*.

**Table 1:** ORF content of pSDH-1 and pSDH-2.

**Plasmid**	**ORF**				**Protein**				
	**Annotation**	**Start**	**End**	**Strand**	**Putative function**	**Closest homolog^*a*^**	**Genomic context^*b*^**	**% identity (aa)/% query cover**	**Accession number**
pSDH-1	*orf1*	74	238	+	Hypothetical	No similarity found	NA	NA	NA
	*orf2*	327	776	+	Hypothetical	No similarity found	NA	NA	NA
	*orf3*	773	940	+	Hypothetical	No similarity found	NA	NA	NA
	*orf4*	1,140	937	−	Hypothetical	No similarity found	NA	NA	NA
	*repA*	2,423	1,470	−	Plasmid replication	Replicase family protein, plasmid pRGRH0347	P	45/97	CRY94758.1
	*orf6*	2,838	2,596	−	Hypothetical	No similarity found	NA	NA	NA
	*mobA*	4,275	2,905	−	Mobilization (relaxase)	Relaxase (*V. parahaemolyticus*)	UD	53/60	KOF22943.1
	*mobC*	4,630	4,265	−	Mobilization	Mobilization protein C (*Moraxella macacae* plasmid pMOMA1)	P	33/97	WP_009502314.1
	*orf9*	3,584	3,270	−	Hypothetical	Hypothetical protein (*V. parahaemolyticus*)	UD	35/80	WP_045625915.1
	*orf10*	3,173	2,844	−	Hypothetical	Two-component sensor histidine kinase (*Azohydromonas australica* DSM 1124)	C	26/48	WP_028999828.1
pSDH-2	*orf1*	243	82	−	Hypothetical	No similarity found	NA	NA	NA
	*orf2*	460	263	−	Hypothetical	Hypothetical protein (*V. tasmaniensis*)	P	57/45	AKN38663.1
	*mipA*	1,391	2,161	+	MltA-interacting protein	MipA/OmpV family protein (*V. cholerae* VCC19)	C	99/99	WP_001078596.1
	*parE*	2,617	2,333	−	Toxin component of TA system, plasmid stabilization/partitioning	ParE/Doc from *Vibrio* sp. FF_304	P	81/100	AKN38163.1
	*parD*	2,846	2,607	−	Antitoxin component of TA system, plasmid stabilization/partitioning	CopG/PhD from *Vibrio* sp. FF_304	P	86/100	AKN38162.1
	*orf6*	3,449	2,991	−	Hypothetical	No similarity found	NA	NA	NA
	*orf7*	3,702	3,857	+	Hypothetical	Hypothetical protein DN41_3524 (*V. cholerae* 1421_77)	UD	73/98	KFD82702.1
	*orf8*	4,123	3,986	−	Hypothetical	No similarity found	NA	NA	NA
	*orf9*	4,157	4,273	+	Hypothetical	No similarity found	NA	NA	NA
	*mobM*	4,368	5,348	+	Mobilization/recombination	Plasmid recombination enzyme family protein (*V. cholerae* HE-09)	UD	98/99	EGS55481.1
	*orf11*	5,560	5,357	−	Hypothetical	No similarity found	NA	NA	NA

a *Or closest homolog with a putative function assigned. In a case where the closest homolog was labeled as putative protein the best significantly close hit with a putative function was used*.

b *Genomic context of the homolog. P, plasmid (or extrachromosomal element); C, chromosome, UD, undetermined*.

NA: not applicable

**Figure 2:**
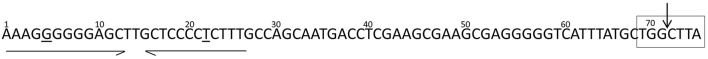
Putative oriT region of pSDH-1. Near-perfect inverted repeats are indicated by arrows. Nucleotide mismatches are underlined. Nucleotides identical to the putative oriT region of ColE1 are boxed and the specific dinucleotide where the relaxase-mediated cleavage occurs (*nic*) in ColE1 is indicated by the arrow.

*orf7* and *orf8* were respectively renamed *mobA* and *mobC*. The protein encoded by *mobA* shares homology with a relaxase protein from *Vibrio parahaemolyticus*, although similarity was observed for only part of the protein sequence. The product of *mobC* shares low similarity (33%) with MobC from pMOMA1, a small cryptic plasmid recently described in *Moraxella macacae* (Whitehouse et al., 2015), but has no conserved domain hits. In various instances (Francia et al., 2004) MobA/MobC proteins have been shown to be part of a relaxosome, a protein complex required for initiation of transfer of conjugative and mobilizable elements. Conjugative elements encode all the functions required for their own transfer from a donor to a recipient cell, such as the proteins encoding the conjugative machinery and the components of the relaxosome. On the other hand, mobilizable elements rely on the conjugative machinery provided by a co-residing conjugative element. In most cases mobilizable elements encode their own relaxase, but some cases of *trans*-acting relaxases have been reported (Daccord et al., 2010; Carraro et al., 2017). The putative MobA protein encoded by pSDH-1 seems to be related to the MOB_HEN_ family of relaxases (Francia et al., 2004), to which the ColE1 relaxase belongs. Although, it shares only low overall similarity with the relaxases of this family, the conserved motifs previously described are perfectly conserved in pSDH-1 MobA, with the exception of proline in motif III. The MOB_HEN_ family relaxases are typically associated with accessory proteins, namely MobC, normally encoded by a gene located directly upstream of *mobA* and one or two other proteins encoded by genes located within the *mobA* coding sequence (Francia et al., 2004). In the case of pSDH-1, we were able to identify a MobC-like protein, encoded by the ORF located directly upstream of *mobA*. However, the two predicted proteins encoded by the ORFs embedded in the *mobA* coding sequence (*orf9* and *orf10*) do not contain any mobilization domains or features. Further analysis of the nucleotide sequence of pSDH-1 revealed a region that is similar to the origin of transfer (oriT) found in plasmids carrying a MOB_HEN_ family relaxase (Figure 2). This region, located 52 bp upstream the MobC translation start site contains a sequence (CTGGCTTA) that is identical to the one found in the ColE1 oriT and which contains the cleavage site (or *nic* site) of ColE1 relaxase. Additionally, the putative oriT of pSDH-1 comprises two nearly perfect inverted repeats. Although the sequence of these inverted repeats differs between pSDH-1 and ColE1, they are located at a similar relative distance from the putative *nic* sites.

### Identification and genomic structure of plasmid pSDH-2

Plasmid pSDH-2 encodes 11 putative ORFs (Figure 1 and Table 1). *orf1, orf6, orf8, orf9*, and *orf11* encode putative proteins of unknown function and do not have any identifiable homologs. *orf2* encodes a putative protein that shares 57% identity on 45% of its sequence with a hypothetical protein from a *Vibrio tasmaniensis* plasmid. *orf3* encodes a protein sharing 99% identity with a membrane protein from *V. cholerae* strain VCC19. Further analysis shows that it contains MipA/OmpV domains as well as a predicted signal peptide (Blastp and SMART). MipA (MltA-interacting protein) is believed to be a scaffolding protein for murein synthesis that mediates assembly of the lytic transglycosylase MltA with the bifunctional transglycosylase/transpeptidase PBP1B, which respectively play a role in synthesis and degradation of peptidoglycan (Vollmer et al., 1999). Furthermore, UVC- and γ- irradiation, as well as starvation, were shown to induce expression of MipA in some strains of *V. alginolyticus* (Ben Abdallah et al., 2010; Abdallah et al., 2012) suggesting a possible role in host cell persistence in the environment. pSDH-2 *orf3* was renamed *mipA*.

*orf4* and *orf5* encode a putative ParD-ParE type II toxin-antitoxin (TA) system. Homologs of both these proteins were found as part of the same TA system on an extrachromosomal element, likely a plasmid, from *Vibrio sp*. FF_304. Type II TA systems act as plasmid stabilization systems. Loss of the plasmid by segregation results in death of the plasmid-free cell, since the toxin is more stable than its cognate antitoxin, a mechanism termed post-segregational killing. BlastP and SMART analysis revealed that the protein encoded by *orf4* contains a parE_toxin domain. A BlastP search showed that the product of *orf5* contains a predicted transcriptional regulator COG3905 domain and SMART analyses further revealed that the N-terminal portion of *orf5* product carries an RHH_1 motif. This ribbon-helix-helix motif is found in the ParD antitoxin of the RP4 ParD-ParE TA system, as well as in many transcriptional repressors, and is believed to mediate the DNA-binding function of ParD (Oberer et al., 2007). The unstructured C-terminal region is believed to be involved in interaction with its cognate toxin, ParE. o*rf4* and *orf5* were respectively renamed *parE* and *parD*.

*orf10*, renamed *mobM*, encodes a protein that shares 98% identity with a plasmid recombination protein from *V. cholerae* HE-09 and carries a SMC_proK_B domain (structural maintenance of chromosomes) at its C-terminal end. The N-terminal region of MobM_pSDH−2_ contains most of the conserved residues found in motifs I, II and III of Mob and Pre proteins of the pMV158 family (Francia et al., 2004). Plasmid recombination proteins such as Pre from plasmid pMV158 (also termed MobM) are relaxases involved in conjugative transfer (Priebe and Lacks, 1989; Francia et al., 2004). BlastP analysis of MobM_pMV158_ reveals that it also carries a C-terminal SMC_prok_B domain. Plasmids carrying a relaxase belonging to the pMV158 superfamily have been shown to be mobilizable by a large number of conjugative plasmids and integrating conjugative elements (also known as conjugative transposons) (Francia et al., 2004) (and references therein).

### Identification of the putative origin of replication of pSDH-2

**Figure 3:**
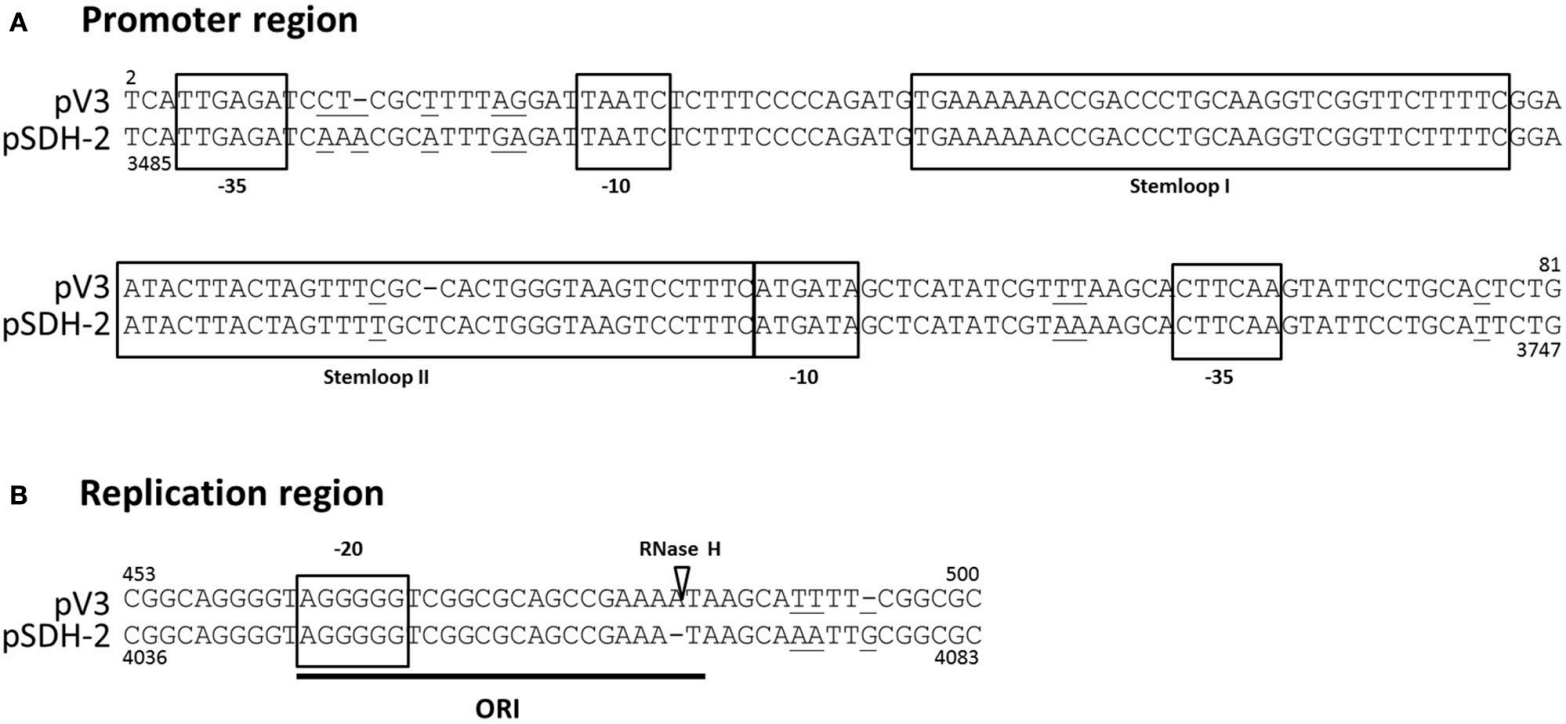
Alignment of putative promoter **(A)** and replication **(B)** regions of pSDH-2 with corresponding regions of the CLV origin of plasmid pV3 (GU272161.1; Pan et al., 2010). The main features of the CLV origin are indicated by boxes and their function is indicated. Nucleotides that differ between the two sequences are underlined. The RNase H cleavage site is indicated by a white triangle. Positions on the nucleotide sequences of pSDH-2 and pV3 are indicated below and above the alignment, respectively.

Comparison of the nucleotide sequence of pSDH-2 with publicly available sequences reveals that it shares no similarity with known plasmids, with the exception of two ca. 500 bp stretches, the first located between *orf2* and *mipA* and the second encompassing *orf7* and half of *orf8*. The first of these regions shares 88% similarity with an intergenic region of a mobilizable plasmid identified by *in silico* analysis in *Vibrio tasmaniensis* ZF-76 clone 102 (KP795524.1) (Xue et al., 2015). The second region shares 97% homology with the replication region of small *Vibrionaceae* plasmids (Figure 3) shown to utilize a ColE1-like replication mechanism (Pan et al., 2010). Like ColE1, these CLV (ColE1-like Vibrionaceae) plasmids encode two constitutively expressed and convergently transcribed RNAs (RNA I and RNA II), which respectively specify their incompatibility and replication initiation determinants (Pan et al., 2010). Studies (Pan et al., 2010; Brantl, 2014; Lilly and Camps, 2015) (and references therein) of the replication of plasmid ColE1 have shown that RNA II is the only plasmid-encoded feature required for initiation of replication. The ~550-bp pre-primer form of RNA II forms a persistent hybrid (R-loop) with the lagging-strand at the site of the origin of replication. Once the R-loop is formed, RNA II is processed by RNase H, producing a 3′-OH end that serves as a primer for leading-strand synthesis. RNA I (108-bp) specifies incompatibility and controls copy number of ColE1 by binding with the RNA II pre-primer and inhibiting R-loop formation. The corresponding region of pSDH-2 contains all the features typically found in CLV plasmids, suggesting it replicates following the ColE1 model of plasmid replication (Figure 3). Furthermore, the regions of pSDH-2, corresponding to the two ColE1 RNAs, are predicted to fold into stem loop structures (Figure S1).

### pSDH-1 and pSDH-2 distribution in haitian isolates

**Figure 4:**
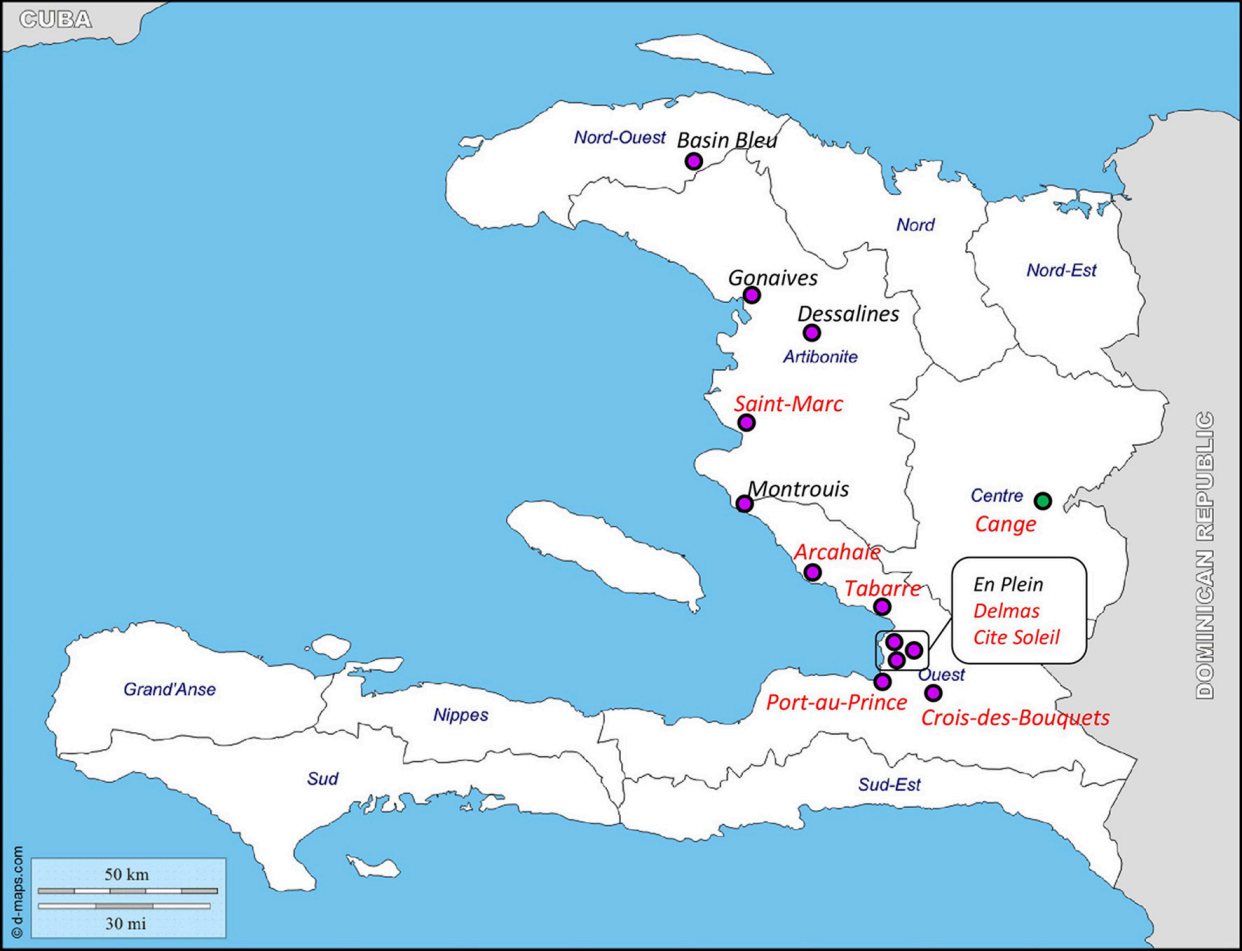
Distribution of pSDH-1 and pSDH-2 in Haiti. Purple and green circles indicate sites where clinical and environmental isolates were collected, respectively, in the Departments of Nord-Ouest, Artibonite, Ouest, and Centre. Red indicates districts where pSDH-1 and/or pSDH-2 were detected (see Table 2). Original figure was downloaded from http://www.d-maps.com/carte.php?num_car=5262&lang=en, according to the website terms and conditions of use.

Two sets of primers specific for each plasmid (Figure 1) were designed to allow screening of a collection of Haitian strains, including *V. cholerae* O1 and *V. cholerae* non-O1/non-O139 isolated from clinical and environmental samples, and *Vibrio* spp. and *Aeromonas* spp. isolated from the same biological samples. pSDH-1 and/or pSDH-2 were predominantly detected in *V. cholerae* non-O1/non-O139 of clinical origin (34 out of 120). However, both plasmids were detected in a *V. cholerae* non-O1/non-O139 environmental isolate from the Cange district (Table 2). Two clinical isolates of *V. cholerae* O1 isolated in Cite Soleil also carried pSDH-1 and/or pSDH-2. 107 isolates did not hold any of the two plasmids. Other *Vibrio* spp. and *Aeromonas* spp. were all negative (data not shown). Both plasmids were geographically widespread in 8 of 13 analyzed districts, with variable prevalence in each Department (Figure 4): 33.7% (*n* = 30) in Ouest, 14.3% (*n* = 4) in Artibonite, 4.7% (*n* = 1) in Centre, and none in Nord Ouest (Table 2). pSDH-2 was the most common element either alone (*n* = 14) or in association with pSDH-1 (*n* = 21). The latter was never detected alone. Presence of the ParD-ParE TA system on pSDH-2 may explain why this plasmid was found more frequently since the TA system would ensure maintenance. Alternatively, pSDH-2 could be mobilized at a higher frequency or by a broader range of helper conjugative elements than pSDH-1.

**Table 2:** Distribution of pSDH-1 and pSDH-2 among clinical *V. cholerae* non-O1/non-O139 and O1 isolated in different Haitian Departments.

**Department (district)**	***V. cholerae*** **non-O1/non-O139**			***V. cholerae*** **O1**		
	**pSDH-1 and pSDH-2**	**pSDH-2^*a*^**	**NP**	**pSDH-1 and pSDH-2**	**pSDH-2^*a*^**	**NP**
**Artibonite**						
Dessalines						2
Gonaïves			4			5
Saint Marc	4		3			10
**Centre** (Cange)		1^*^	21^*^			
**Nord Ouest** (Bassin Bleu)			1			2
**Ouest**						
Arcahaie		2				4
Cite Soleil				1	1	6
Croix-des	3	2				5
-Bouquets						
Delmas	4	2	3			1
En Plein						2
Montrouis						4
Port-au-	8	6	5			25
Prince						
Tabarre	1		2			2
Total (142)	20	13	39	1	1	68

a *Detection of only pSDH-2*.

NP: no plasmid detected

* *V. cholerae non-O1/non-O139 isolates from Cange were of environmental origin*.

## Conclusion

In this study, we report two previously unknown plasmids prevalent in *V. cholerae* in the early phase of the 2010 Haitian cholera outbreak. The genome content of these plasmids suggests self-mobilization and, at least in the case of one of them, a TA system for plasmid stabilization through post-segregational killing. pSDH-1 and pSDH-2 enrich the list of small cryptic plasmids circulating in *Vibrio* spp. in the aquatic (Bidinost et al., 1999; Powers et al., 2000; Zhang et al., 2007) and clinical environments and whose role in bacterial fitness or pathogenicity merits further investigation.

## Author contributions

Data analysis: DC, GG, and NH. Strain screening: DC. Single-cell genomics: RS. Plasmid assembly and annotation: GG, SC, and RS. Contributed materials and reviewed the paper: MP, AH, and RC. Manuscript writing: DC and GG. All authors discussed, read, contributed to, and approved the final manuscript.

### Conflict of interest statement

SC, NH, and RC were employed by company CosmosID Inc. The other authors declare that the research was conducted in the absence of any commercial or financial relationships that could be construed as a potential conflict of interest.
