# Supplementary figures and images for: Characterization of Two Cryptic Plasmids Isolated in Haiti from Clinical Vibrio cholerae Non-O1/Non-O139
Source: Front Microbiol. 2017 Nov 23;8:2283. doi: 10.3389/fmicb.2017.02283 (PMC5703827; doi:10.3389/fmicb.2017.02283)

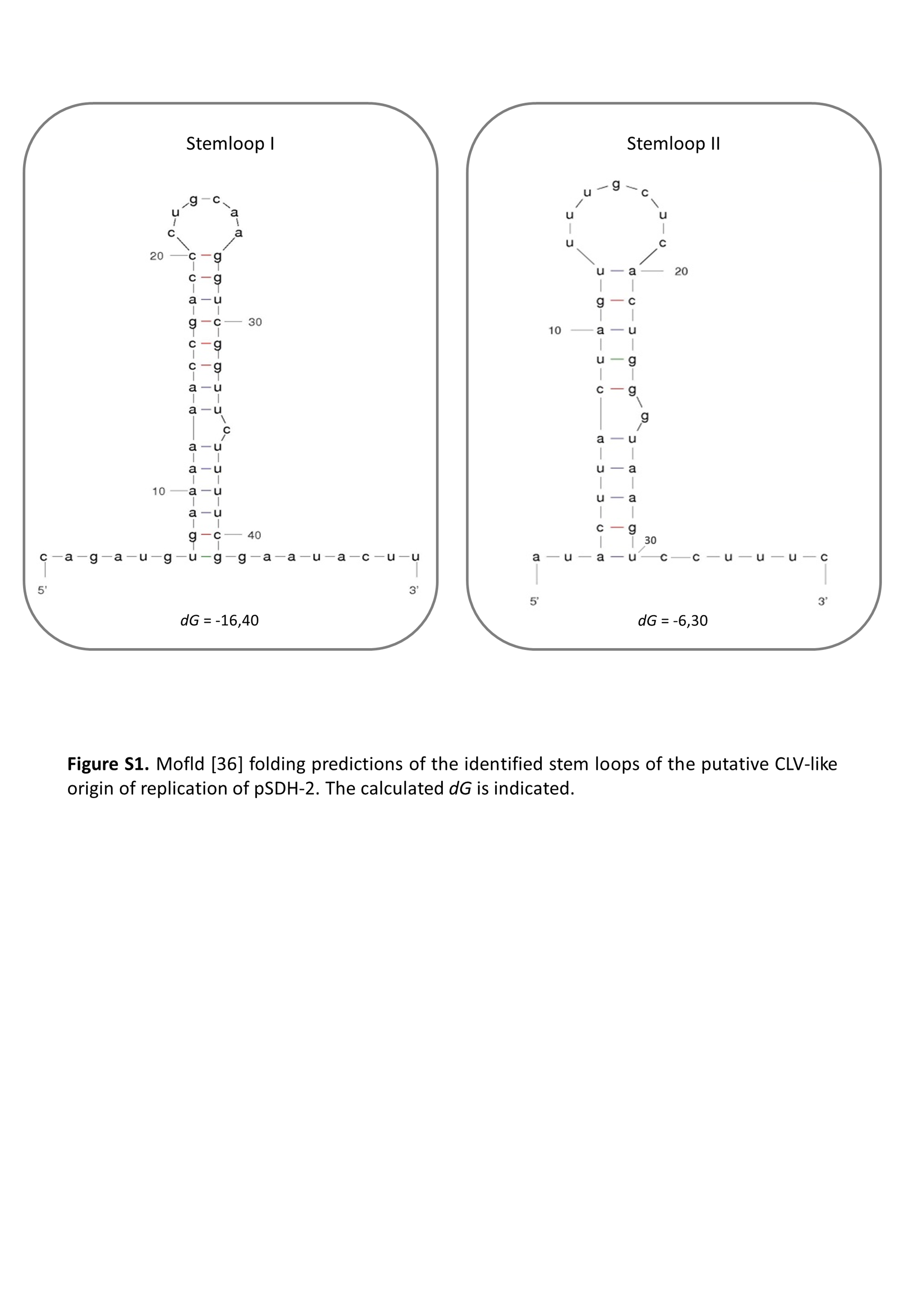

Supplement: Supplementary file 1 [file Image1.tif]
